# Clinic flow for STI, HIV, and TB patients in an urban infectious disease clinic offering point-of-care testing services in Durban, South Africa

**DOI:** 10.1186/s12913-018-3154-2

**Published:** 2018-05-11

**Authors:** Katrina J. Stime, Nigel Garrett, Yukteshwar Sookrajh, Jienchi Dorward, Ntuthu Dlamini, Ayo Olowolagba, Monisha Sharma, Ruanne V. Barnabas, Paul K. Drain

**Affiliations:** 1 Centre for the AIDS Programme of Research in South Africa (CAPRISA), University of KwaZulu-Natal, Durban, South Africa; 2 School of Medicine, University of Washington, Seattle, USA; 3 MRC-CAPRISA HIV-TB Pathogenesis and Treatment Research Unit, Durban, South Africa; 4 School of Nursing and Public Health, Discipline of Public Health Medicine, University of KwaZulu-Natal, Durban, South Africa; 5 Prince Cyril Zulu Communicable Disease Centre, eThekwini Municipality, Durban, South Africa; 6 Department of Global Health, Schools of Medicine and Public Health, University of Washington, Seattle, USA; 7 Department of Medicine, School of Medicine, University of Washington, Seattle, USA; 8 Department of Epidemiology, School of Public Health, University of Washington, Seattle, USA

**Keywords:** Point-of-care, South Africa, Implementation science, Differentiated care, HIV, STI, TB

## Abstract

**Background:**

Many clinics in Southern Africa have long waiting times. The implementation of point-of-care (POC) tests to accelerate diagnosis and improve clinical management in resource-limited settings may improve or worsen clinic flow and waiting times. The objective of this study was to describe clinic flow with special emphasis on the impact of POC testing at a large urban public healthcare clinic in Durban, South Africa.

**Methods:**

We used time and motion methods to directly observe patients and practitioners. We created patient flow maps and recorded individual patient waiting and consultation times for patients seeking STI, TB, or HIV care. We conducted semi-structured interviews with 20 clinic staff to ascertain staff opinions on clinic flow and POC test implementation.

**Results:**

Among 121 observed patients, the total number of queues ranged from 4 to 7 and total visit times ranged from 0:14 (hours:minutes) to 7:38. Patients waited a mean of 2:05 for standard-of-care STI management, and approximately 4:56 for STI POC diagnostic testing. Stable HIV patients who collected antiretroviral therapy refills waited a mean of 2:42 in the standard queue and 2:26 in the fast-track queue. A rapid TB test on a small sample of patients with the Xpert MTB/RIF assay and treatment initiation took a mean of 6:56, and 40% of patients presenting with TB-related symptoms were asked to return for an additional clinic visit to obtain test results. For all groups, the mean clinical assessment time with a nurse or physician was 7 to 9 min, which accounted for 2 to 6% of total visit time. Staff identified poor clinic flow and personnel shortages as areas of concern that may pose challenges to expanding POC tests in the current clinic environment.

**Conclusions:**

This busy urban clinic had multiple patient queues, long clinical visits, and short clinical encounters. Although POC testing ensured patients received a diagnosis sooner, it more than doubled the time STI patients spent at the clinic and did not result in same-day diagnosis for all patients screened for TB. Further research on implementing POC testing efficiently into care pathways is required to make these promising assays a success.

## Background

Many clinics in Southern Africa have long waiting times [1–3]. This is of particular concern in infectious disease clinics, where the availability of new treatments has led to increased demand for healthcare services and overcrowded facilities. Long waiting times have been linked to low patient satisfaction, skipped appointments, poor medication compliance, and low health worker morale, resulting in inefficient treatment in places where the burden of infectious diseases is highest [4–8].

Recent literature has suggested a number of reasons for long waiting times, including staff shortages, poor clinic organization, and laboratory delays [9, 10]. Point-of-care (POC) tests, which can be performed by non-laboratory personnel within the clinical setting, may help reduce waiting times. POC tests are being increasingly adopted in low- and middle-income countries because they provide timely information for clinical management and reduce the need for follow-up visits [11, 12]. POC tests allow staff to receive results without waiting for sample transport and central laboratory processing, patients to receive timely care, and laboratory personnel to focus their time on tests that must be performed in the laboratory.

Despite these advantages, the shift toward POC diagnostics may have positive or negative effects on clinic flow. For example, POC CD4 testing for HIV reduced time to antiretroviral therapy (ART) initiation in Mozambique, and GeneXpert® POC tuberculosis (TB) testing reduced time to multi-drug resistant TB diagnosis in South Africa [13–15]. In contrast, POC testing can also increase waiting times, lengthen clinic visits, and place extra demands on staffing and space [16]. POC TB testing in India produced significant delays and POC antenatal syphilis testing in Mongolia was time-consuming for providers [17, 18]. Thus, a primary barrier to implementing POC tests may be challenges with clinical integration rather than diagnostic accuracy [19]. A better understanding of baseline clinic flow, health worker attitudes toward POC tests, and potential barriers to clinical integration could ensure that POC tests do not significantly disrupt patient flow.

Our objectives were to perform a baseline assessment of clinic flow at a large public healthcare clinic in Durban, South Africa, that is in the process of implementing POC testing. We evaluated the clinic flow prior to implementation of POC testing for HIV viral load (VL), during implementation of POC testing for sexually transmitted infections (STIs), and after implementation of POC testing for TB. To evaluate clinic flow during implementation of POC testing for STIs, time in motion data was collected concurrently for patients undergoing standard-of-care syndromic management and patients undergoing POC diagnostic management as part of a cohort study.

We predicted that patients spent a large amount of time at the clinic and that implementation of POC tests increased waiting times since patients required more steps to complete a visit.

## Methods

### Clinic description

We conducted a time in motion study at the Prince Cyril Zulu Communicable Disease Centre (PCZ CDC), the largest government outpatient HIV, TB, and STI treatment facility in Durban, South Africa. PCZ CDC is located in Durban’s central business district, adjacent to a busy transport hub, and serves patients from the greater Durban and surrounding areas. The clinic has three clinicians, fourteen professional nurses, three enrolled nurses, two laboratory technicians, two radiographers, and two administrators to care for over 500 TB, STI, and HIV-positive patients per business day. At the clinic, all medical services are free of charge to patients.

STIs were treated based on patient-reported symptoms using algorithms developed by the World Health Organization [20]. During clinical appointments, nurses gathered histories, performed physical examinations as needed, prescribed and administered medications, provided notes for partner therapy, and completed patient charts. As part of a cohort study to assess diagnostic POC testing with expedited partner therapy, a subset of PCZ CDC patients underwent testing for *Chlamydia Trachomatis* and *Neisseria Gonorrhoeae* with GeneXpert® CT/NG assay (Cepheid, Sunnydale, California, US), *Trichomonas vaginalis* by OSOM® Rapid Trichomonas Test (Sekisui Diagnostics, Lexington, MA, US), and microscopy for candida and bacterial vaginosis. These patients went through the same queues as the standard-of-care patients, but saw research nurses for consent, STI testing, and treatment.

HIV screening was performed with a two HIV antibody POC test algorithm (InTec, Xiamen, China, and Abon, Hangzhou, China) provided by the South African Department of Health and, if discordant, confirmed with a laboratory enzyme-linked immunosorbent assay. Three professional nurses each managed HIV care and ART dispensing for 45 to 50 HIV-positive patients per day. One professional nurse managed an alternative “fast-track” queue, which allowed clinically stable patients who were virologically suppressed on ART to visit the clinic less frequently and avoid the standard professional nurse queues. These patients visited the clinic twice instead of six times per year and saw a special nurse who managed only uncomplicated patients. During clinical appointments, nurses gathered histories, performed clinical examinations and adherence counseling as needed, and distributed ART. After initiating HIV treatment, patients were recommended to receive VL testing every six months in the first year and annually thereafter. Three nurse phlebotomists each collected samples from a mean of 65 patients per day. Three-quarters of these included samples for VL testing at an off-site laboratory. Of note, four laboratories processed all VL tests from the KwaZulu-Natal province, which involved collecting, transporting, processing, and returning approximately 100,000 VL tests each month. VL results were available online approximately seven days after collection through an electronic laboratory platform (InterSystems TrakCare Lab™) and were generally reviewed by nurses at the patient’s next visit.

The clinic performed POC TB testing using an on-site 16-module GeneXpert® machine (Xpert MTB/RIF; Cepheid, Sunnyvale, CA), which processed samples within 90 min in the National Health Laboratory Services site laboratory. A single technician usually batch-tested up to 16 samples in two to three daily runs, processing up to 48 samples per day. Patients who tested positive saw an enrolled nurse to open a TB file and a professional nurse or doctor to initiate treatment. Patients who tested negative saw a professional nurse for further management. Patients renewing TB medications saw one of the five professional nurses who also provided care for STI patients.

### Data collection

#### Patient observations for time in motion

Data was collected from June through August of 2016. For the time in motion data collection, patients were observed at different times of day on multiple days of the week to help reduce the sampling bias. Every third patient was selected for data collection to generate a cohort that may be representative of the clinic population. Patients who presented for multiple services (e.g., HIV and TB care) during their visit were excluded because they skipped certain queues. When collecting data, we first asked patients what time they arrived at the clinic. In the few cases where patients were unsure, we estimated the arrival time by asking someone who arrived at a similar time as the patient. Clinic staff recorded the time each chart was retrieved. The research team collected data on all other steps. We gathered time data through observation to avoid interrupting clinic flow with research activities. To evaluate clinic flow during implementation of POC testing for STIs, time in motion data was collected concurrently for patients undergoing standard-of-care syndromic management and patients undergoing POC diagnostic management as part of a cohort study.

To understand why many patients arrived at the clinic very early in the morning, we asked patients who arrived prior to 7 a.m., “Why did you come to the clinic so early?” This question was translated by a Zulu-speaking nurse and asked while patients waited in line before clinic opening.

#### Staff interviews

To gather qualitative evidence on clinic flow and POC testing, we conducted semi-structured interviews with PCZ CDC staff. We selected 20 clinic staff, including six nurses, two physicians, five laboratory technicians (two from PCZ CDC staff, three from the POC STI research team), five administrators (two from PCZ CDC staff, three from the POC STI research team), and two security guards to encompass many aspects of clinic operations. The research team interviewed clinic staff individually in English. Staff were encouraged to express their opinions on aspects of the clinic that worked well, aspects that could be improved, and the use of POC tests at the clinic. We summarized interviews using basic thematic analysis.

### Data analyses

We compiled data in Excel and used descriptive statistics to summarize individual data for STI, HIV, and TB treatment services. Times were described in terms of mean, median, standard deviation, and range.

## Results

**Fig. 1:**
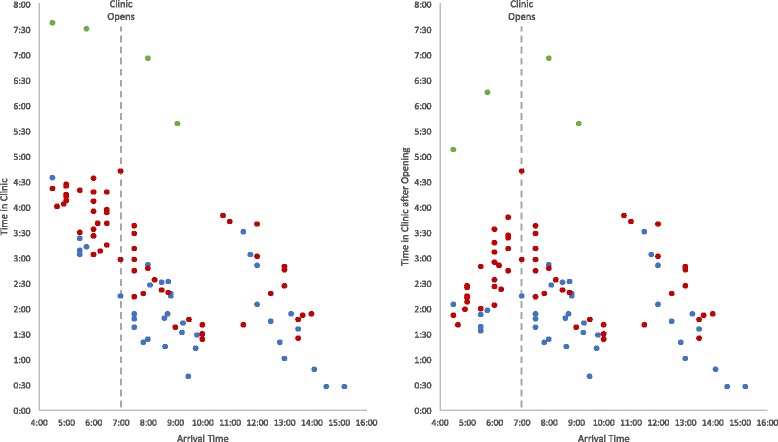
Total time spent at the clinic by arrival time. Time in clinic is reported in hours and minutes (hours:minutes). Waiting time before the clinic opened is included in the graph to the left and excluded in the graph to the right. Blue represents STI patients undergoing syndromic management, red represents HIV patients, and green represents patients being screened for TB. Four patients screened for TB returned the following day for results, resulting in much longer clinic visits (not shown)

Among 121 participants observed, 48 patients presented for STI care, 56 presented for HIV care, and 17 presented for TB care. Of the 112 patients with recorded arrival times, thirty-three (29%) arrived prior to the clinic opening at 7 a.m. and waited a mean of 1:29 (hours:minutes) before the doors opened. Patients who arrived before 7 a.m. tended to spend more total time at the clinic. However, they spent similar amounts of time at the clinic after the doors opened to those who arrived later (Fig. 1). Reasons for arriving early included: to avoid long queues later in the day; to get to work on time; to avoid disclosing his or her HIV status at work; and to make the cut-off time for the blood sample courier to the central laboratory. Patients were accepted at the chart collection desk until 3:30 p.m. with no patient cap for services. Nurses stayed until the last patient was seen at around 4 p.m.

### Patients with STIs

**Fig. 2:**
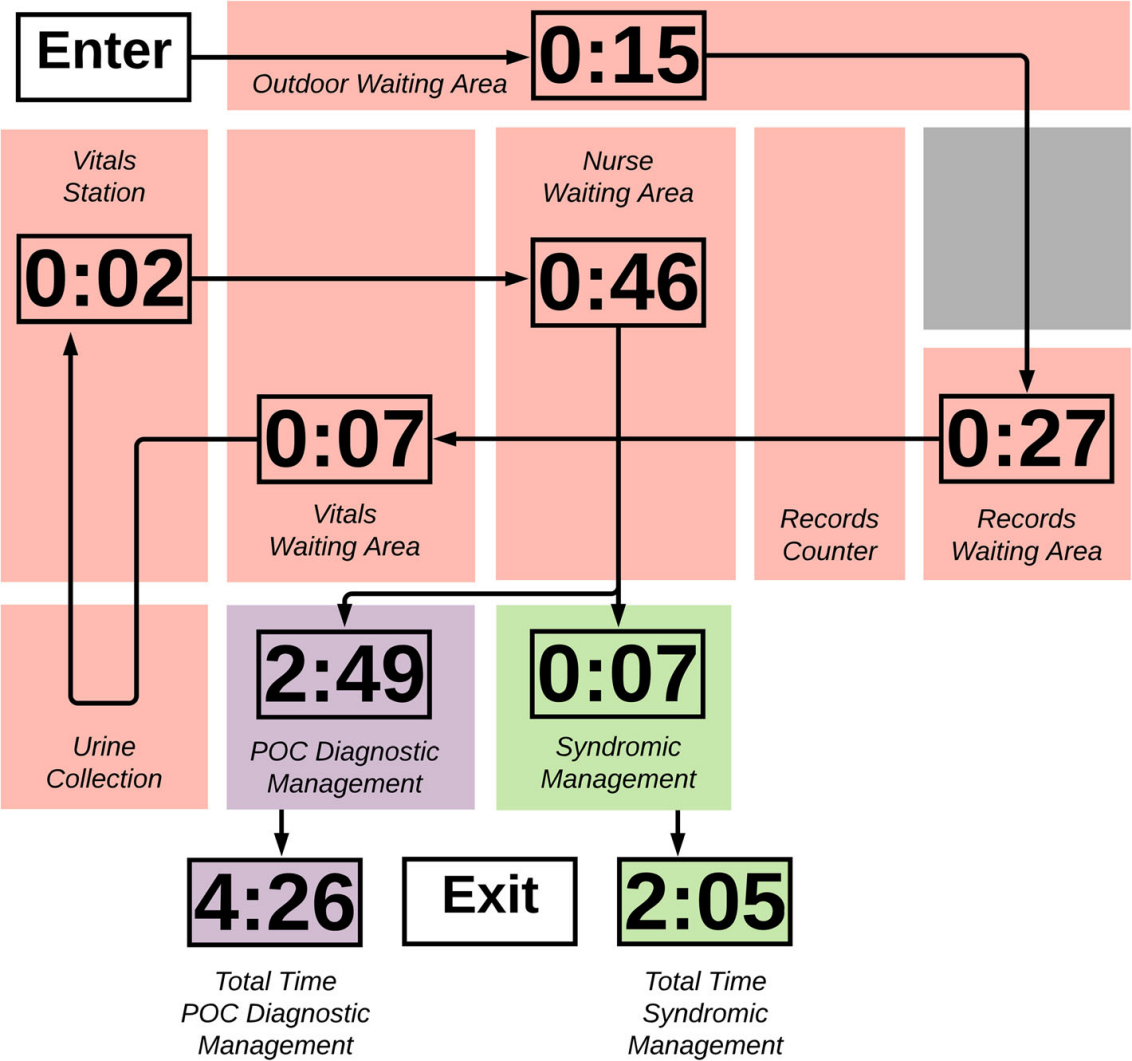
STI patient flow map for syndromic versus POC diagnostic management. Times are reported in hours and minutes (hours:minutes). Red boxes are areas involved in STI patient care, the green box represents exam rooms for syndromic management, the purple box is the exam room for POC diagnostic management, and the grey boxes are not directly involved in STI patient care. Map is not to scale

STI patients were seen by five professional nurses who each provided care to approximately 50 patients with STIs or TB per day. Among 39 syndromically managed STI patients, the mean total visit duration was 2:05 (hours:minutes), and the mean clinical appointment duration was seven minutes (Table 1). On average, patients spent 6% of their waiting time receiving care. Patients waited in four queues, and the longest steps were waiting to collect the patient chart (mean 27 mins) and waiting to see a nurse (mean 46 mins) (Fig. 2). These two steps comprised 58% of total visit time.

**Table 1:** Time and motion data for patients receiving STI services

	INTERVAL	N	Mean (SD)	Median	Min	Max
Common Times	Time waiting before clinic opening	39	0:15 (0:37)	0:00	0:00	2:30
	Time waiting for chart	38	0:27 (0:21)	0:20	0:01	1:19
	Time waiting for vitals	9	0:07 (0:03)	0:07	0:03	0:15
	Time obtaining vitals	15	0:02 (0:01)	0:03	0:01	0:06
	Time waiting for nurse	13	0:46 (0:33)	0:40	0:04	1:44
Syndromic Management	*Time with nurse*	*37*	*0:07 (0:04)*	*0:06*	*0:01*	*0:20*
	*Total time in clinic*	*39*	*2:05 (0:42)*	*1:48*	*0:28*	*4:35*
	*% of time receiving care*	*–*	*6%*	*–*	*–*	*–*
POC Diagnostic Management	*Time with nurse*	*9*	*2:49 (0:28)*	*2:52*	*2:06*	*3:38*
	*Total time in clinic* ^a^	*–*	*4:26 (1:01)*	*4:02*	*–*	*–*
	*% of time receiving care*	*–*	*64%*	*–*	*–*	*–*

Times are reported in hours and minutes (hours:minutes). The italicized rows indicate data specific to patients undergoing standard-of-care syndromic management or POC diagnostic management. POC patients waited in the same queues as standard-of-care patients and were recruited while waiting to see a nurse

^a^Because entry times were not observed for these patients, total time in clinic was calculated by summing the common times with the time with the nurse. N, min, and max are not reported

One professional and one enrolled nurse managed approximately five patients per day who underwent diagnostic management with STI POC testing as part of a research study. Among nine patients in this pathway, there was a mean additional visit time of 2:49 of which the longest step was running the GeneXpert® samples (2:12). When added to the time collecting charts and measuring vitals in the syndromic arm, estimated total visit time for the STI POC testing pathway was 4:56. While the clinical visit accounted for 64% of the total time, much of this time was spent waiting for results.

### Patients with HIV

**Fig. 3:**
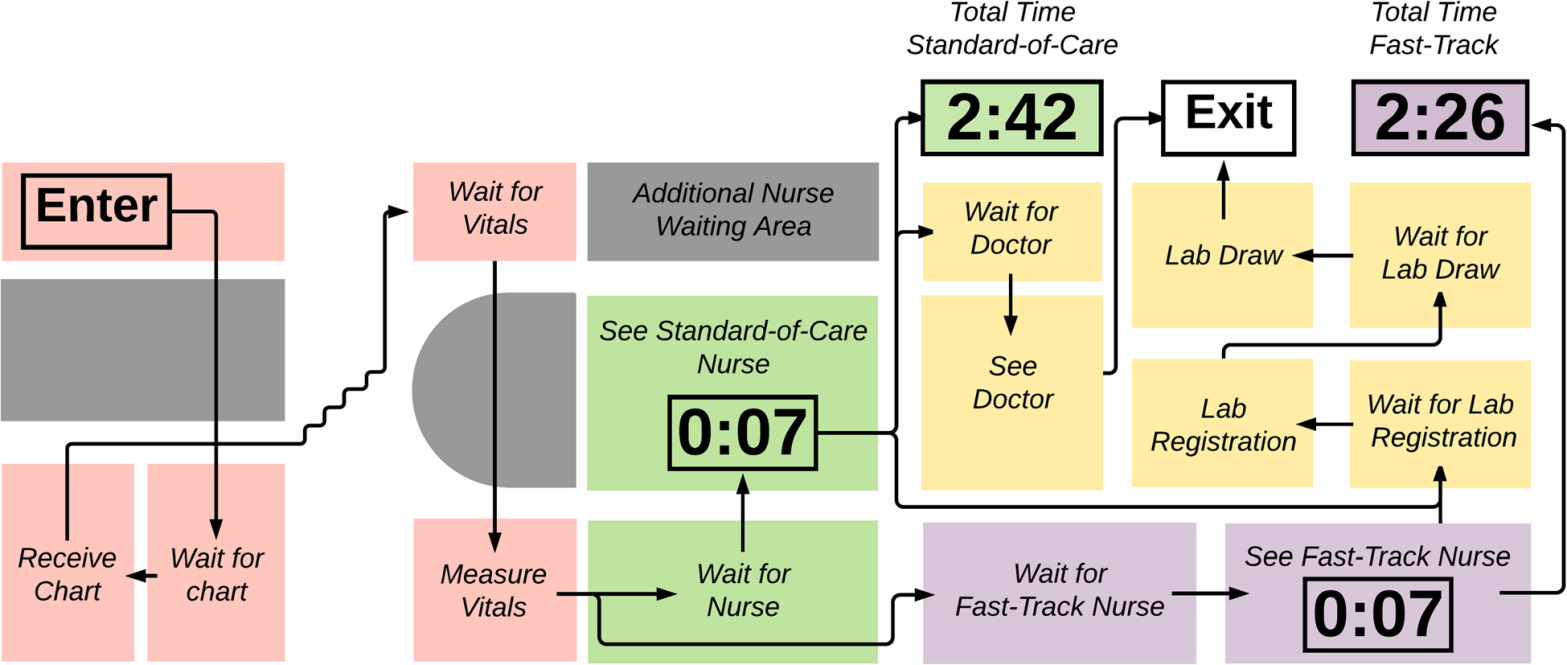
HIV patient flow map for standard-of-care and fast-track queues. Times are reported in hours and minutes (hours:minutes). Red boxes are areas involved in HIV patient care, green boxes are standard-of-care waiting areas and exam rooms, purple boxes are fast-track waiting areas and exam rooms, yellow boxes are possible pathways after standard or fast-track, and grey boxes are not directly involved in HIV patient care. Map is not to scale

Patients renewing HIV medications waited in four queues, and patients due for blood tests or requiring a doctor’s visit waited in up to seven queues (Fig. 3). Among 27 patients, the mean total visit time for stable HIV patients waiting in four queues was 2:42 (hours:minutes). The mean clinical appointment duration with a professional nurse was seven minutes, which was 4% of the total visit time (Table 2).

**Table 2:** Time and motion data for patients receiving HIV services in standard-of-care and fast-track queues

	INTERVAL	N	Mean (SD)	Median	Min	Max
Standard-of-care	Time with nurse	27	0:07 (0:03)	0:07	0:03	0:15
	Total time in clinic	27	2:42 (0:44)	2:46	1:25	3:50
	% of time receiving care	–	4%	–	–	–
Fast-track	Time with nurse	29	0:07 (0:03)	0:08	0:01	0:14
	Total time in clinic	28	2:26 (0:49)	2:24	0:14	4:43
	% of time receiving care	–	5%	–	–	–

Times are reported in hours and minutes (hours:minutes)

Patients in the alternative “fast-track” waited in four queues. Among 28 patients, the mean total visit time was 2:26. The mean clinical appointment duration with a professional nurse was seven minutes, equivalent to the normal HIV care pathway, and equating to 5% of total visit time.

### Patients being screened for TB

**Fig. 4:**
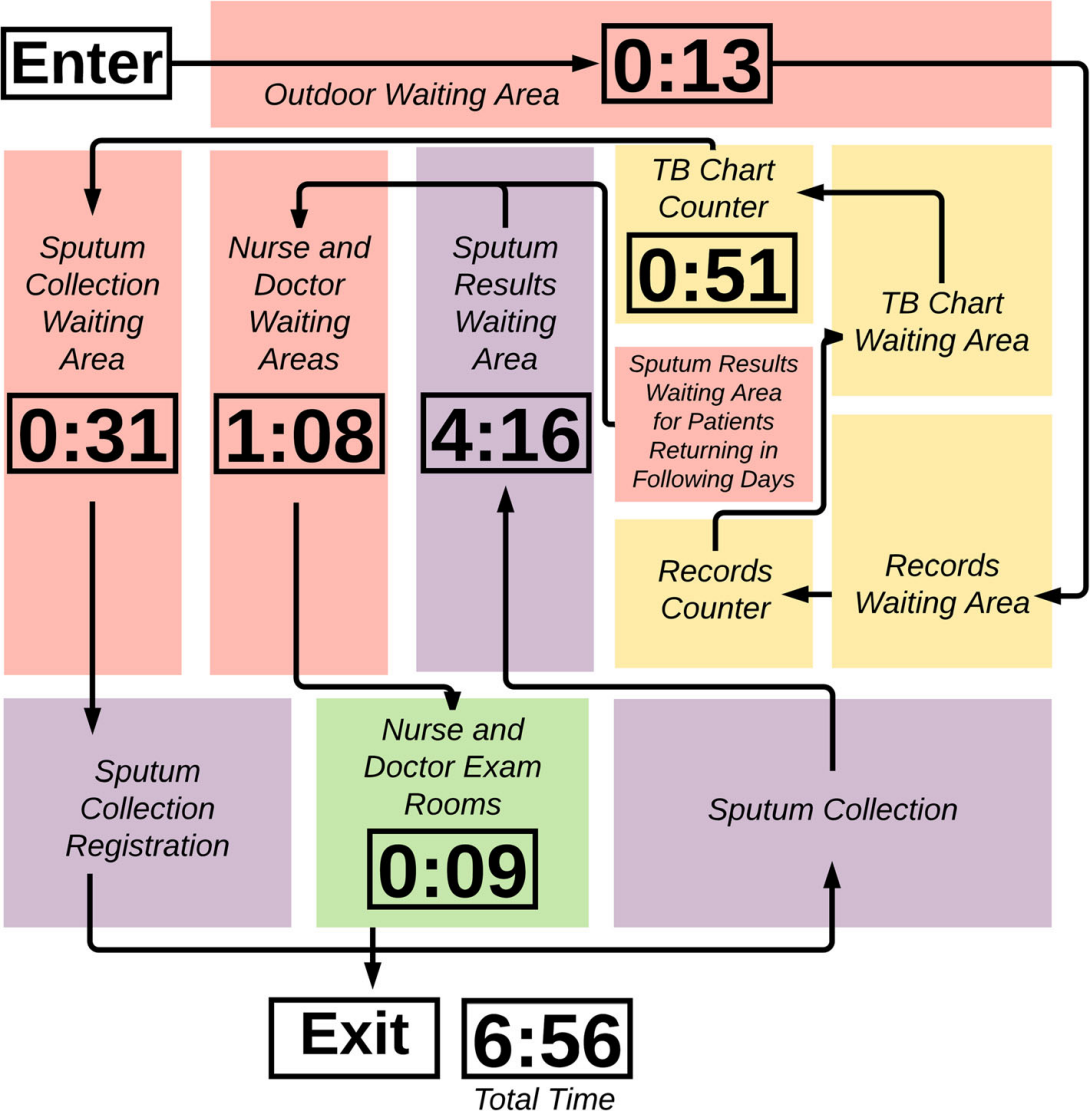
TB patient flow map. Times are reported in hours and minutes (hours:minutes). Yellow boxes are all included in the 0:51 waiting time, purple boxes are all included in the 4:16 waiting time, the green box is exam rooms, and red boxes are other areas involved in TB patient care. Map is not to scale

Patients being screened for TB waited in six queues. Among the 17 patients observed, the four who completed testing and treatment initiation took a mean of 6:56 (hours:minutes) from arrival to exit (Table 3). The mean time patients spent at the clinic before collecting a sputum sample was 1:06. After collecting the sample, patients spent a mean of 4:16 before receiving results, which includes the 90-min GeneXpert® assay run time (Fig. 4). Four out of ten patients were asked to return the following day for results because their samples were collected after 1 p.m., which staff considered too late in the day to complete the whole process.

**Table 3:** Time and motion data for patients receiving TB screening services

INTERVAL	N	Mean (SD)	Median	Min	Max
Time waiting before clinic opening	17	0:13 (0:40)	0:00	0:00	2:30
Time waiting for chart	9	0:51 (0:22)	0:50	0:11	1:13
Time waiting for sputum collection	9	0:31 (0:09)	0:32	0:11	0:43
Time waiting for sputum results	6	4:16 (0:24)	4:12	3:56	4:52
Time waiting for nurse or doctor	4	1:08 (0:33)	1:02	0:35	1:53
Time with nurse or doctor	4	0:09 (0:04)	0:08	0:06	0:16
Total time in clinic	4	6:56 (0:54)	7:13	5:39	7:38
% of time receiving care	–	2%	–	–	–

Patients being screened for TB gathered charts with STI patients and then went to a separate queue for sputum collection. Times are reported in hours and minutes (hours:minutes). Four of ten patients screened for TB were asked to return the following day for results and are not included in the total time in clinic

### Staff interviews

Three main topics were discussed during staff interviews: clinic efficiency, staffing and space, and POC tests in the health system. First, there was consensus that operations could be run more efficiently. However, staff identified different sources of inefficiency within the system. One administrator who worked at the chart desk described difficulties finding patient charts since they were often in the wrong place. She also described how filing VL results from the regional laboratory in patient charts took a large portion of her time. One professional nurse saw fewer patients so that he could direct traffic in the HIV clinic. He explained that patients often did not know where to go since the signs on the floor had worn off.

Some staff raised concerns about staffing and space. According to one doctor, “There are 10,000 to 12,000 people on ART. One medical officer can see a maximum of 4,800 stable patients per year. However, when you add in children, patients with high VLs, co-infected patients, and patients with drug-induced liver injuries, that number goes down to 1,200 per year. There is not enough clinic space for all the doctors that are needed to cover the patients.” Furthermore, the study team observed that roughly two to three staff were on sick leave each day, placing greater strain on the system. Some staff were also concerned that the “test and treat” HIV care model (introduced in South Africa after the completion of this study) would increase patient volume. These challenges contributed to staff’s hesitancy to accept new POC tests, which could potentially further increase workload.

As a whole, staff were in favor of expanding POC tests, though they mentioned both benefits and challenges. According to one doctor, “In rural South Africa, patients with high VLs aren’t seen for several weeks because of the time it takes to receive VL results from the national laboratory. These places would benefit most from VL POC testing.” Another doctor noted the following regarding the POC TB test: “For small clinics it can take one to two weeks to get TB results. However, for [PCZ CDC], they just wait around four hours for GeneXpert.” Other staff highlighted the challenges facing implementation of POC tests considering clinic flow. According to a PCZ CDC nurse, “If a patient’s TB sputum sample isn’t collected by around 10 a.m., it’s too late and the patient has to come back the next day for results.” Another PCZ CDC doctor noted that “It’s important that POC tests are actually implemented as POC tests. If it ends up taking 24 hours to get results, then it makes no difference if the test is done at the PCZ CDC or at the central lab.”

## Discussion

This busy urban clinic in Durban, South Africa, which cares for up to 500 patients per business day, had several queues to navigate and, therefore, patients had long visits for STI management and HIV care. Time in motion data showed that patients spent a large amount of time at the clinic, but a small amount of time with providers. POC TB tests often did not provide same-day results. Qualitative data revealed several factors contributing to these delays: large patient volumes, long POC assay times, and staff shortages.

When compared with patients at other infectious disease clinics in Sub-Saharan Africa, patients at PCZ CDC spent similar amounts of time at the clinic. However, patients at PCZ CDC spent a smaller percentage of time receiving services than waiting for care. Surveys of several HIV clinics in Uganda found that patients spent between 1:17 and 4:34 at the clinic, compared to 2:42 for HIV patients at PCZ CDC. The same studies found patients spend 15 to 46% of their time with a provider, whereas HIV patients at PCZ CDC spent 4 to 5% of their time with a provider [1–3]. One plausible explanation for this difference is that each South African nurse treated around 50 patients per day – much greater than the suggested nurse workload of 35 patients per day recommended by the South African District Health Barometer [21]. Doctors also had a large workload of 25 to 35 patients per day. This limited the amount of time PCZ CDC providers could spend with each patient.

Our findings are consistent with the literature on queuing theory, which suggests that networks with nodes of varying efficiencies create bottlenecks and increase overall waiting times [22]. Networks with many nodes can also create confusion about where to go in the absence of clear communication or sign posts and cause patients to exit care while waiting between rooms [13, 22, 23]. In our setting, nodes such as the chart counter and nurse waiting area had high patient-to-staff ratios, while other nodes such as the vitals station had low ratios, resulting in long waiting times. Additionally, overlapping queues with long walkways between them caused confusion among patients. Integrating visit steps into a single consultation could improve care delivery.

Our study is limited by its observational nature and sample size. Although we collected data on multiple providers at multiple times of the day without interfering with patient flow, more accurate estimations would have been obtained by gathering data on every patient that entered the clinic at each step. Our methods are an alternative that could more easily scale to other South African clinics, while still providing valuable insights. It is also possible that being observed led to behavioral change or alteration in activity. This concern was mitigated by having a limited number of research staff in the clinic and recording from afar when possible. Finally, our analysis may have been more comprehensive if we had a larger sample size. However, this was unlikely to significantly alter the trend or direction of our findings because we observed multiple staff, visits, and days for each service to capture a representative sample. The qualitative data supported the quantitative findings. Clinic staff who reviewed our results, including sub-group analyses, indicated that the data reflected their experience of clinic flow and provided good reasons for the long visits.

Considering that services are largely standardized across public healthcare clinics, other government clinics may face challenges similar to those described in this study. In addition, many clinics in Southern Africa are set up similar to PCZ CDC, with geographical separation and individual queues for charting, vitals, clinical visits, POC testing, and consultations [24]. While these shared characteristics may present similar challenges, other characteristics such as clinic size, staffing, and budget may differ across clinics and present unique challenges.

Our study also relates to the tradeoffs between syndromic and POC diagnostic management of STIs. Advantages of syndromic management include low price, quick turnaround, and no requirement for laboratory technology. Disadvantages include failure to detect asymptomatic cases, which account for the majority of infections, as well as poor positive predictive value, which can lead to overtreatment and overuse of antibiotics [25]. Our results highlight the relative efficiency of syndromic management, which took a mean of seven minutes per patient compared to 2:49 (hours:minutes) for POC diagnostic management.

Regarding TB management, we found that few people were tested and initiated on treatment in the same day. Similar delays have been identified in other settings where, despite using a two-hour assay, patients were initiated on treatment two to three days after testing due to manpower requirements and backlogs [26, 27]. The single laboratory technician at PCZ CDC prepared and ran up to 48 TB tests per day, demonstrating a shortage of manpower. The 90-min assay time further contributed to backlogs. Until these delays are fixed, some patients may prefer to leave and return another day rather than wait 6:56 (hours:minutes). However, a majority of our study population preferred to wait at the clinic either because they lived far away or because they were too sick to leave and return.

## Conclusion

In summary, we have described long patient waiting times for short clinical visits for patients seeking STI, HIV, and TB services at a large public healthcare clinic in Durban, South Africa. We have also described the extent of POC test implementation at this clinic. As new POC tests become available for STI, HIV, and TB diagnostics and monitoring, they may result in even longer visits if implementation challenges are not addressed. Our study supports the idea that resources should be allocated to improving patient flow and care models in addition to developing better and faster POC tests.

## Abbreviations

ART: Antiretroviral therapy
HIV: Human immunodeficiency virus
NHLS: National Health Laboratory Service
PCZ CDC: Prince Cyril Zulu Communicable Disease Centre
POC: Point-of-care
STI: Sexually transmitted infection
TB: Tuberculosis
VL: Viral load
